# Engineered photoresponsive biohybrids for tumor therapy

**DOI:** 10.1002/SMMD.20220041

**Published:** 2023-03-10

**Authors:** Xiaocheng Wang, Yazhi Sun, Daniel Wangpraseurt

**Affiliations:** ^1^ Department of NanoEngineering University of California San Diego San Diego California USA; ^2^ Scripps Institution of Oceanography University of California San Diego San Diego California USA

**Keywords:** biomimetic materials, engineered biohybrid materials, phototherapy, tissue engineering, tumor therapy

## Abstract

Engineered biohybrids have recently emerged as innovative biomimetic platforms for cancer therapeutic applications. Particularly, engineered photoresponsive biohybrids hold tremendous potential against tumors due to their intriguing biomimetic properties, photoresponsive ability, and enhanced biotherapeutic functions. In this review, the design principles of engineered photoresponsive biohybrids and their latest progresses for tumor therapy are summarized. Representative engineered photoresponsive biohybrids are highlighted including biomolecules‐associated, cell membrane‐based, eukaryotic cell‐based, bacteria‐based, and algae‐based photoresponsive biohybrids. Representative tumor therapeutic modalities of the engineered photoresponsive biohybrids are presented, including photothermal therapy, photodynamic therapy, synergistic therapy, and tumor therapy combined with tissue regeneration. Moreover, the challenges and future perspectives of these photoresponsive biohybrids for clinical practice are discussed.

## Graphical abstract

In this review, the latest progresses of engineered photoresponsive biohybrids for tumor therapy are summarized. Representative engineered photoresponsive biohybrids are highlighted including biomolecule‐associated, cell membrane‐based, eukaryotic cell‐based, bacteria‐based, and algae‐based photoresponsive biohybrids. Such engineered photoresponsive biohybrids provide biomimetic platforms for diverse cancer therapeutic applications, including photothermal therapy, photodynamic therapy, synergistic therapy, and tumor therapy combined with tissue regeneration.

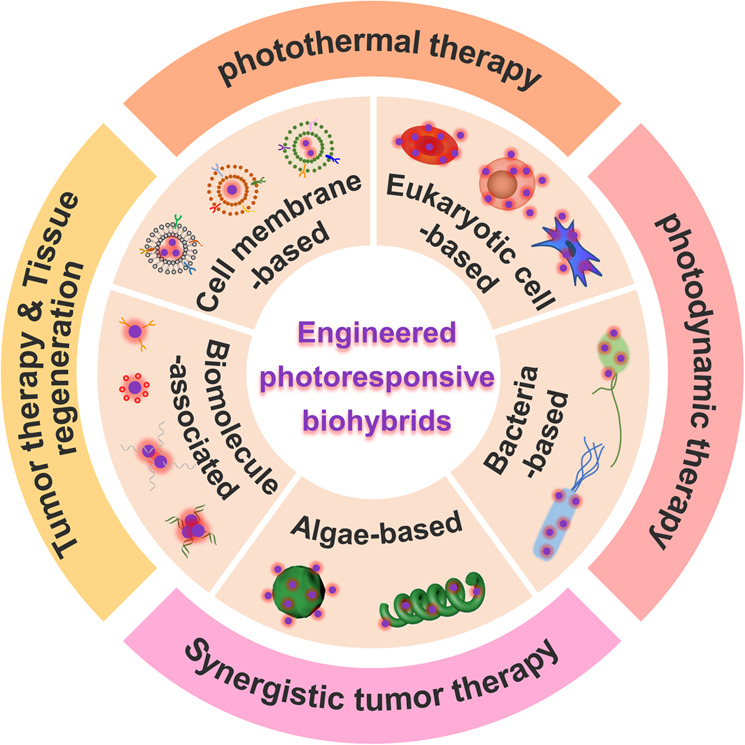

1

**Key points**

- The development of engineered photoresponsive biohybrids and their latest progresses for tumor therapy are summarized.
- Different types of engineered photoresponsive biohybrids are highlighted.
- The range of tumor therapeutic modalities of engineered photoresponsive biohybrids is summarized.
- The potential for future development of engineered photoresponsive biohybrids for tumor therapy is discussed.

## 2 INTRODUCTION

Cancer remains one of the most intractable global healthcare problems, accounting for estimated 10 million deaths worldwide in 2020 or approximately one in six deaths.^1^ The three main tumor therapeutic strategies include surgical excision, radiotherapy, and chemotherapy.^2^, ^3^, ^4^ These conventional methods commonly suffer from several shortcomings, including poor therapeutic efficacies, serious side effects, multidrug resistance, tumor recurrence, or metastasis after treatment.^5^, ^6^, ^7^, ^8^ Over the past decades, various new strategies have been emerged for tumor therapy, such as phototherapy (e.g., **photodynamic therapy** [PDT, **see Glossary**] and **photothermal therapy** [PTT]), immunotherapy, chemodynamic therapy, sonodynamic therapy, magnetic hyperthermia, and gene therapy.^9^, ^10^, ^11^ In phototherapy, PTT typically employs the **photothermal conversion agents** (PTA) to generate heat upon near‐infrared (NIR) irradiation to induce localized thermal damage to tumor tissues, while PDT utilizes the **photosensitizers** (PS) to absorb light energy to generate toxic **reactive oxygen species** (ROS) that result in localized chemical damage to the target lesions.^9^, ^12^, ^13^, ^14^ Owing to its inherent advantages of external light control and minimal invasiveness, phototherapy has been regarded as an efficient and convenient approach for tumor ablation for numerous cancer indications.^15^

As the key components of phototherapy, a variety of photoresponsive agents (including PTA and PS) have been explored, such as noble metal nanoparticles (e.g., Au, Ag, and Pt), carbon‐based nanocomposites (e.g., carbon quantum dots, carbon nanotubes, and graphene oxides), semiconductor nanocrystals (e.g., transition‐metal oxide or sulfide), organic molecules (e.g., indocyanine green [ICG], porphyrin, chlorin e6 [Ce6], metal‐organic frameworks), and semiconducting polymer‐based nanoparticles.^13^, ^16^, ^17^, ^18^, ^19^ Most photoresponsive agents have strong optical absorption of visible lights (400–700 nm) or NIR lights (700–1350 nm).^15^ The control of light placement and the accumulation of photoresponsive agents in the targeted tumor tissues are believed to minimize off‐target toxicity to surrounding tissues.^20^ Unfortunately, the limited selectivity of these agents for tumor tissues necessitates the use of high doses to ensure a significant therapeutic effect, and the accumulation of the agents in non‐malignant tissues may cause undesired side effects.^21^ For example, most inorganic materials like noble metal and transition‐metal sulfide nanoparticles are non‐biodegradable and cannot be effectively excreted by the kidneys, leading to a high risk of systemic toxicity.^22^, ^23^ Although organic agents like cyanine dyes and conjugated polymers have good biocompatibility, biodegradability, and low toxicity, their clinical applications in the human body are still hampered by the potential long‐term safety concerns.^16^ Thus, it presents a great challenge for phototherapy to develop innovative photoresponsive platforms with negligible side effects and excellent tumor therapeutic efficacy.

Recently, photoresponsive biohybrids have gained much attention in widespread applications, including biosensors, biodevices, and biomedical engineering.^24^, ^25^ Biohybrid systems typically employ biological organisms (e.g., bacteria and algae) into artificial material systems, allowing for the design of biomimetic materials with intrinsic self‐assembly and/or self‐replication capabilities.^26^, ^27^ Notably, **engineered photoresponsive biohybrids** may be a novel and promising approach for highly efficient cancer therapy due to a range of advantages. Compared to synthetic photoresponsive materials, biomimetic biohybrids could possess enhanced treatment efficiency considering their innate biological properties, including prolonging the blood circulation time, escaping immune systems from clearance, and delivering of therapeutic drugs to the tumor targets.^28^, ^29^, ^30^ Biohybrids further benefit from the intrinsic self‐assembly and/or self‐replication capabilities of living organisms. As such, the fabrication of biohybrid systems might be sustainable and easily scalable.^31^, ^32^, ^33^ The combination of synthetic and biological components facilitates the design of biohybrid materials with multiple functions, such as tumor‐targeting, effective drug delivery, multimodal imaging, and synergistic tumor therapy.^34^, ^35^, ^36^ Biohybrid systems can be tailored for biocompatibility with mammalian cells and can thus reduce side effects commonly observed with traditional materials/drugs. They further show excellent therapeutic efficacy to tumor tissues,^37^, ^38^, ^39^ thus facilitating the desirable bench‐to‐clinic translation.

**FIGURE 1:**
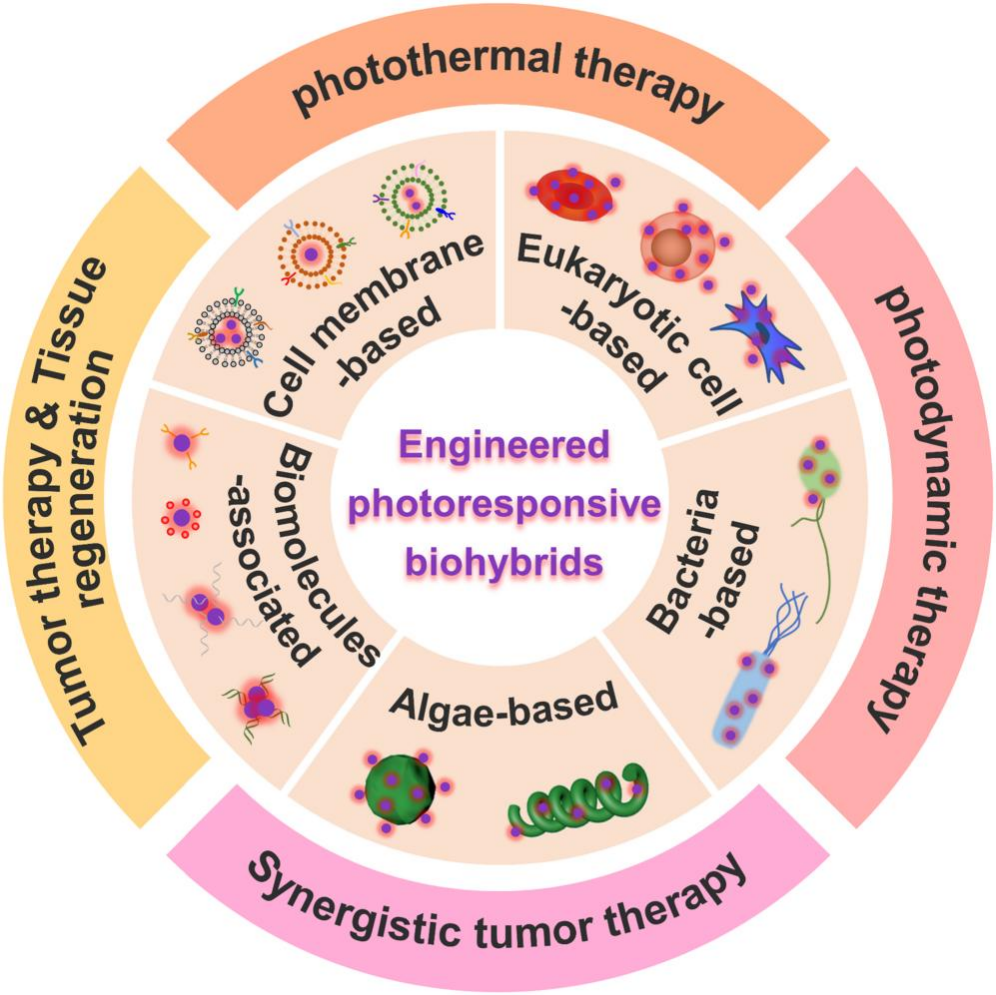
Overview of engineered photoresponsive biohybrids for tumor therapy.

Therefore, research on engineered photoresponsive biohybrids as cancer therapeutic platforms has rapidly progressed in recent years. Here, we systematically summarize the design concept, engineering aspects, and therapeutic strategies of engineered photoresponsive biohybrids for cancer treatment (Figure 1). We first introduce the therapeutic potential and construction methods of such engineered biohybrids and then classify them into biomolecules‐associated, cell membrane‐based, eukaryotic cell‐based, bacteria‐based, and algae‐based photosensitive biohybrids. We then summarize the range of tumor therapeutic modalities of engineered photoresponsive biohybrids, including PTT, PDT, synergistic therapy, and tumor therapy combined with tissue regeneration. Finally, we discuss the potential for future development of engineered photoresponsive biohybrids for tumor therapy.

## 3 DEVELOPMENT OF ENGINEERED PHOTORESPONSIVE BIOHYBRIDS

### 3.1 Tumor therapeutic potential of engineered photoresponsive biohybrids

The successful treatment of malignant tumors remains a great challenge considering their multifactorial physiology (including various tumor sites, volume, stage, and metastasized tumors) and pathological complexity of solid tumors (including dysregulated metabolism, disordered vasculatures, reduced pH value, and hypoxic microenvironment).^40^, ^41^, ^42^, ^43^ The metabolic demands of malignant cells in tumor centers cannot be fulfilled due to insufficient availability of O_2_ and nutrients, resulting in prolonged hypoxia and necrosis. Moreover, with the rapid proliferation of malignant cells, the growing tumor volume increases the distance of the tumor core to adjacent vasculature, thus decreasing O_2_ and nutrient transports to the tumor centers.^44^ The disordered vasculatures render the tumor core inaccessible to all molecular/nanoparticle‐based therapies, because these methods primarily rely on the delivery of therapeutic agents into tumors via the **enhanced permeability and retention** effect.^45^ This issue is a severe limitation for many standard therapeutic approaches (e.g., radiotherapy and chemotherapy) as well as recent nanoparticle‐based therapeutic strategies (e.g., PTT and PDT).^40^, ^46^ Therefore, more effective and smarter therapeutic strategies are highly required for individual tumor microenvironments.

As aforementioned, current existing photoresponsive agents suffer from their poor biocompatibility/biodegradability, limited tumor‐targeting and tumor‐specific accumulation, short blood circulation time, and low therapeutic efficiency.^20^, ^46^, ^47^ More recently, engineered photoresponsive biohybrids have been developed for advanced phototherapy to overcome the complex biological barriers to reach tumor regions.^20^ For example, cell membrane‐coated particles have been frequently used for improving circulation time, tumor‐targeting, and immune stimulation in cancer treatment.^48^, ^49^, ^50^, ^51^, ^52^, ^53^, ^54^, ^55^ Eukaryotic cells like erythrocytes and macrophages have been used as carriers to effectively cross biological barriers and deliver photoresponsive agents to hypoxic tumor sites.^28^, ^56^, ^57^, ^58^ Microorganisms, including bacteria and algal cells, can also be utilized as delivery vehicles of PS into the hypoxic tumors to improve therapeutic effects.^38^, ^59^, ^60^, ^61^ Therefore, it is anticipated that engineered photoresponsive biohybrids could inherit the characteristics from their hybrid components, and provide multifunctional platforms for enhanced tumor therapy.

### 3.2 Engineering strategies of photoresponsive biohybrids

**FIGURE 2:**
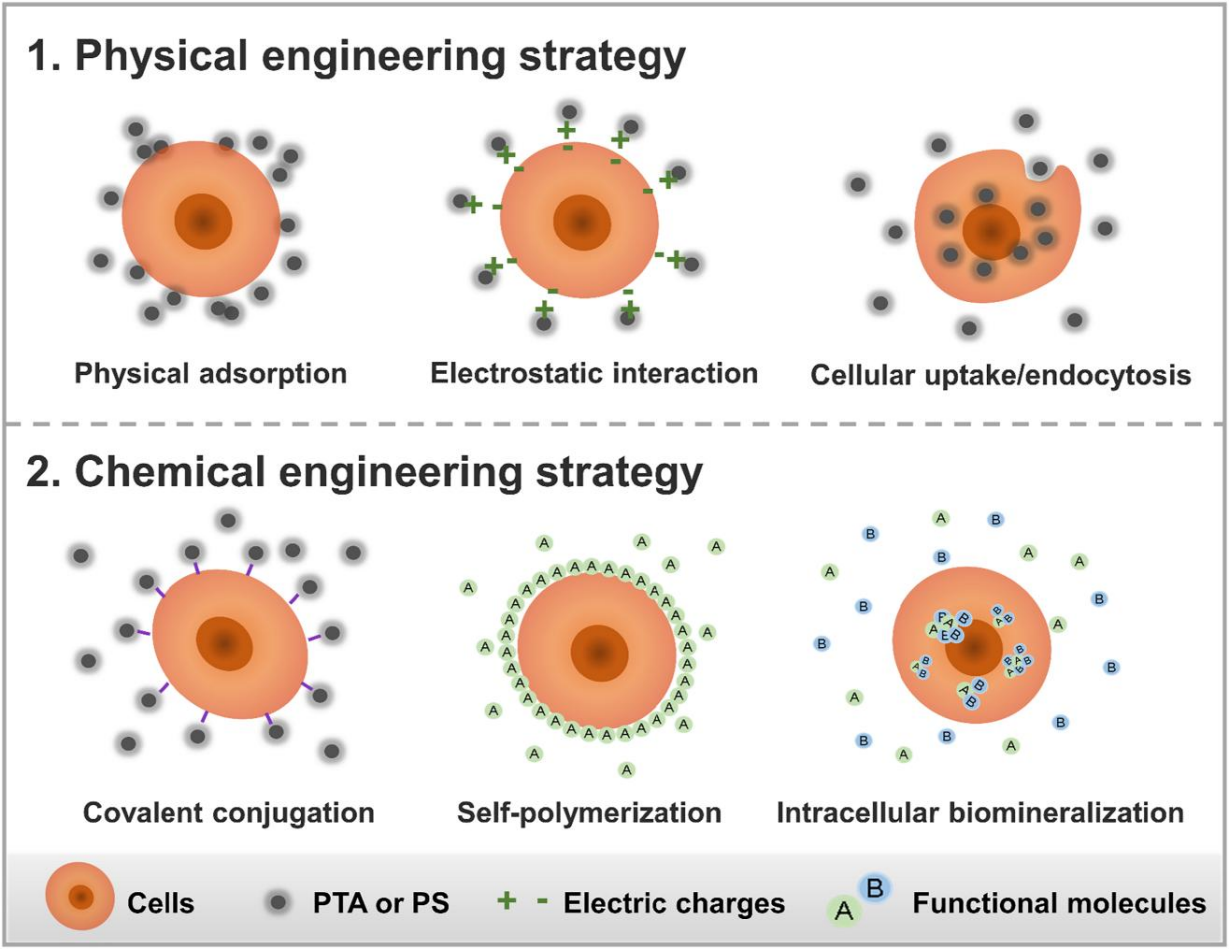
Schematic illustration of representative engineering strategies of photoresponsive biohybrids.

Basically, engineered photoresponsive biohybrids are artificially engineered photoresponsive materials consisting of a bioactive and a structural component. The bioactive part of the biohybrid could consist of cells or bioactive molecules, and the structural part could also be of either biological or non‐biological origin. At present, the engineering strategies of photoresponsive biohybrids mainly include physical and chemical engineering strategies (Figure 2 and Table 1). Physical engineering approaches usually undergo a physical mixing or incubation process to fabricate photoresponsive biohybrids via physical adsorption or encapsulation,^4^, ^62^, ^63^, ^64^, ^65^, ^66^, ^67^, ^68^, ^69^ electrostatic interaction,^3^, ^29^, ^34^, ^35^, ^39^, ^70^, ^71^, ^72^ cellular uptake/**endocytosis**,^30^, ^58^, ^73^, ^74^, ^75^, ^76^, ^77^, ^78^, ^79^, ^80^ hypotonic dialysis,^56^ diffusion,^37^, ^60^, ^81^, ^82^ or electroporation,^83^ etc. For example, PS with positive surfaces could be deposited on the negatively charged cell surface via electrostatic interaction.^84^ The photosensitive nanoparticles could be encapsulated within intact cells via hypotonic dialysis^56^ or cellular uptake.^57^, ^85^, ^86^ By contrast, chemical engineering approaches involve chemical bonds and covalent binding, such as covalent conjugation,^2^, ^38^, ^61^, ^87^, ^88^, ^89^, ^90^ self‐polymerization^36^, ^91^ or **biomineralization**,^32^, ^92^, ^93^, ^94^, ^95^ **bioorthogonal reaction**,^96^ and so on. In one example, Tang et al. first biotinylated both ZnF16Pc‐loaded ferritin (P‐FRT) and red blood cells (RBCS) using biotin‐X‐NHS (Calbiochem) and then crosslinked them with neutravidin to conjugate P‐FRT to RBCs.^96^ The living materials could also be modified with the in‐situ‐synthesized photoresponsive agents via a self‐polymerization or biomineralization process.^33^, ^59^ It is noted that both physical and chemical methods can only provide a temporary or short‐term modification. Comparatively, the physical modification may be more biocompatible and friendly to host cells than chemical methods considering the potential cytotoxicity to host cells imposed by chemical reactions.^97^

**TABLE 1:** Representative fabrication methods for the engineered photoresponsive biohybrids.

Classification	Methods	Characteristics	Advantages	Disadvantages	Examples
Physical engineering strategy	Physical adsorption/encapsulation	Noncovalent interactions	Simple operation, mild condition, and no toxic reagents	Limited loading efficiency and low stability	Cell membrane coated microalgae^65^ and/or nanoparticles^67^, ^68^, ^69^
	Electrostatic interaction	Electrostatic force	Simple operation, mild condition, and relative high aqueous stability	Limited loading efficiency, unspecific interactions, and potential toxicity	Peptide and amino acids modified photosensitizers,^3^ engineered bacteria/algae with nanoparticles^29^, ^34^, ^35^
	Cellular uptake/endocytosis	Nanomaterials internalized into living host cells	Specificity, low toxicity, and tumor targeting efficacy inhered from host cells	Low‐therapeutic agent loading and potential cellular toxicity of nanoagents	Nanoagents internalized into eukaryotic cells^58^, ^73^, ^75^ or bacteria^30^, ^80^
Chemical engineering strategy	Covalent conjugation	Formation of a covalent linkage	Specific interactions, stability, and long‐lasting therapeutic effects	Complex operations, multiple steps, limited loading capacity, potential cytotoxicity to host cells imposed by chemical reactions	Salmonella bacteria^2^, ^38^ or cyanobacteria cells^61^ conjugated with photosensitizers
	Self‐polymerization	Formation of large polymer or massive molecules	Simple operation, mild condition, and stability	Limited material choice, reduced cell viability, and biofunctions after modification	Salmonella bacteria coated with polydopamine^36^ and endogenous protoporphyrin X synthesis in lymphocytes^91^
	Biomineralization	Deposition of mineral within or outside the cells of living organisms.	Mild condition, well‐controllable immobilization, and morphological stability	Limited material choice, time‐consuming reactions, reduced cell viability, and biofunctions after modification	Au nanoparticles embedded throughout in microalgae cells,^32^ self‐mineralized bacterium with Pd nanoparticles,^93^ and calcium phosphate or silica‐modified microalgae^94^, ^95^

### 3.3 Representative types of engineered photoresponsive biohybrids

Here, we classify the current engineered photoresponsive biohybrids based on their biological building blocks, including biomolecule‐associated, cell membrane‐based, eukaryotic cell‐based, bacteria‐based, and microalgae‐based photosensitive biohybrids.

#### 3.3.1 Biomolecule‐associated photoresponsive biohybrids

Biomolecules (e.g., proteins, peptides, and nucleic acids) are fundamental to all living cells, making them suitable building blocks for biohybrid materials.^98^, ^99^, ^100^ Compared to synthetic polymers, natural biomolecules have the capacity to self‐assemble, are suitable for structural templating, and are biocompatible and biodegradable.^100^, ^101^ Moreover, the biomolecules may offer fascinating benefits in tumor therapy, such as improved drug loading efficiency, specific tumor‐targeting effects, controlled drug release, and powerful immune modulation.^4^, ^52^, ^102^, ^103^ Therefore, the development of proteins‐/peptides‐/RNA‐/DNA‐based photoresponsive biohybrids has received increased interest for advanced tumor therapy.^3^, ^63^, ^88^, ^90^, ^104^, ^105^, ^106^, ^107^, ^108^

**FIGURE 3:**
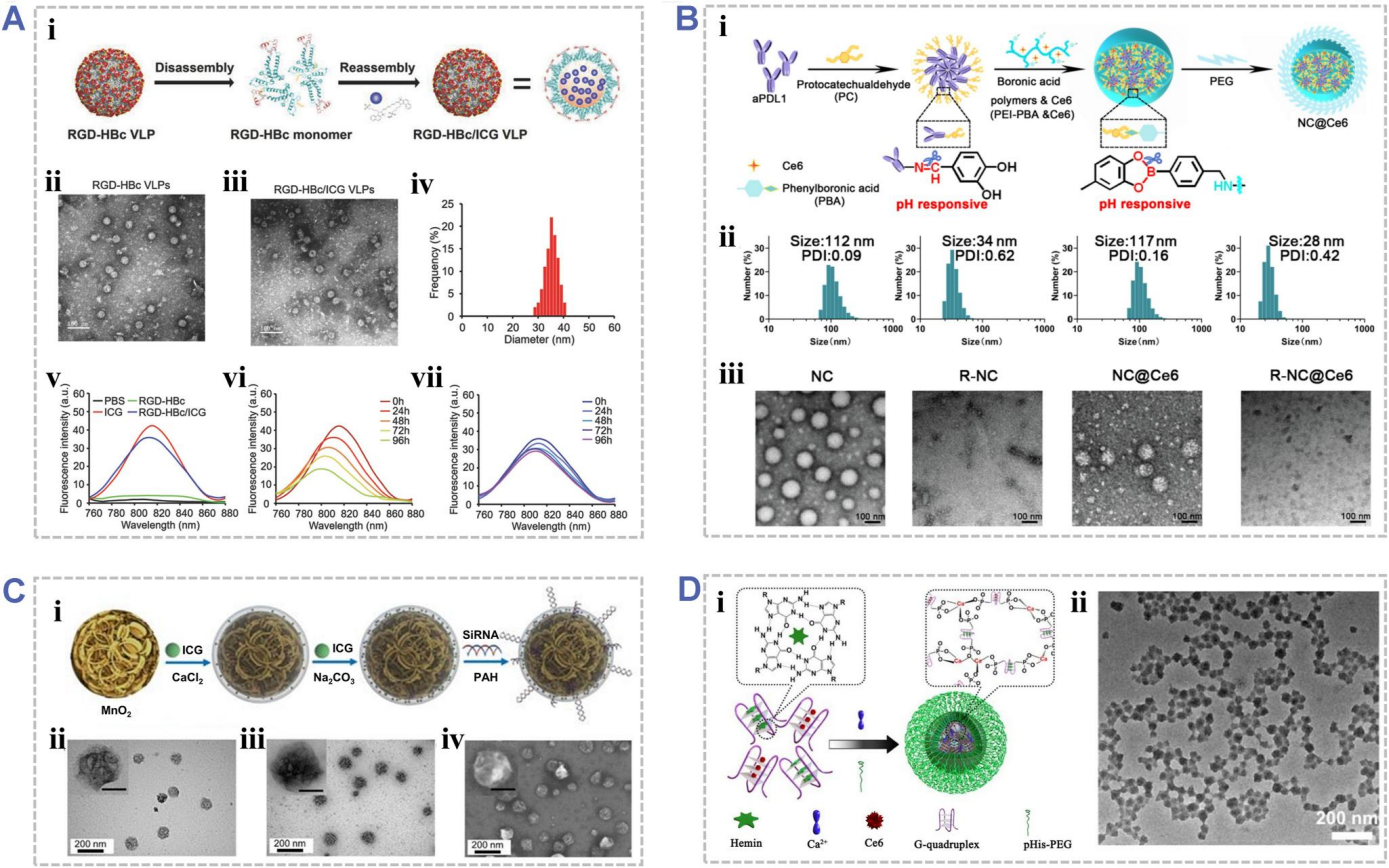
Design of representative biomolecules‐associated photoresponsive biohybrids. (A) Design of a protein‐based nanocomplex (RGD‐HBc/ICG) by incorporating the ICG into an fusion protein (hepatitis B core protein remolded with tripeptide Arg‐Gly‐Asp, RGD‐HBc): (i) Schematic illustrating the preparation procedure of RGD‐HBc/ICG VLP; (ii, iii) TEM images of RGD‐HBc VLPs before (ii) and after (iii) ICG encapsulation; (iv) Diameter distribution of RGD‐HBc/ICG particles; (v) Fluorescence spectra of RGD‐HBc/ICG in the aqueous solution; (vi, vii) Changes of absorption and fluorescence spectra of free ICG (vi) and RGD‐HBc/ICG (vii) in the aqueous solution during 96 h. Reproduced with permission.^3^ Copyright 2018, John Wiley and Sons. (B) Design of a size/charge/targeting changeable nano‐booster (NC@Ce6) by co‐loading the anti‐programmed death‐ligand 1 (aPDL1) and photosensitizer (Ce6) into the acid‐responsive nanocomplex (NC): (i) Schematic illustrating the fabrication procedures of NC@Ce6; (ii) DLS measurement and (iii) TEM images of NC and NC@Ce6, showing that the NC particle size was reduced from 112 nm (pH 7.4) to a tumor penetration favorable size (34 nm) under weakly acidic conditions (pH 6.0). Reproduced with permission.^90^ Copyright 2022, Elsevier. (C) Design of by a biohybrid nanoplatform of Mn@CaCO_3_/ICG@siRNA: (i) Schematic illustration of the synthetic route of Mn O_2_@CaCO_3_/ICG@siRNA; (ii) TEM image of MnO_2_ nanoparticles; (iii) TEM and (iv) SEM images of MnO_2_@CaCO_3_/ICG@siRNA. Reproduced under terms of the CC‐BY license.^105^ Copyright 2019, The Authors, published by Ivyspring International Publisher. (D) Design of DNA nanostructure‐based nanoscale coordination polymers (CACH‐PEG NPCs): (i) Schematic illustration for synthesis of CACH‐PEG NPCs. (ii) TEM image of CACH‐PEG NCPs. Reproduced with permission.^106^ Copyright 2018, American Chemical Society.

For instance, the tripeptide Arg‐Gly‐Asp (RGD), a well‐known tumor‐homing ligand, has been used as a building block in a tumor‐targeting platform. Shan et al. first fabricated tumor‐targeting RGD‐hepatitis B core protein virus‐like particles (HBc VLP), which were then used as carriers to encapsulate the PS ICG through a disassembly/reassembly method (Figure 3A).^3^ The obtained RGD‐HBc/ICG biohybrids not only maintained the original PTT/PDT effects of ICG, but also showed improved stability, long‐term body retention, and tumor‐specific accumulation as compared to free ICG. In another case, Zang et al. developed a size/charge/targeting changeable nano‐booster (denoted as NC@Ce6) for amplifying PDT/immunotherapy (Figure 3B).^90^ The NC@Ce6 was prepared by incorporating the PS Ce6 and anti‐programmed death‐ligand 1 (aPDL1) into a PH‐responsive nanocomplex (NC). The NC showed a reduced size and was positively charged in the presence of the acidic tumor microenvironment, which facilitated a controlled release and efficient sufficient delivery of Ce6 and aPDL1 to the tumor site.

In another example, Liu et al. designed a multifunctional **theragnostic** nanoplatform (MnO_2_@CaCO_3_/ICG@siRNA) by entrapping the MnO_2_ core with a pH‐response cover layer of CaCO_3_ and ICG, which were further functionalized with PD‐L1 siRNA via electrostatic interaction for Figure 3C.^105^ Under acid tumor conditions, the two inorganic materials (MnO_2_ and CaCO_3_) were decomposed into Mn and Ca ions by consuming H^+^ ions while generating O_2_ and CO_2_, which could relieve the tumor microenvironment and promote the release and diffusion of ICG. Moreover, the large amounts of enriched O_2_ in local area facilitated the generation of single oxygen, resulted in enhanced PDT effects against tumor cells. By loading PD‐L1 siRNA on the nanoprobes, the Mn@CaCO_3_/ICG@siRNA could inhibit tumor immune resistance for efficient immunotherapy in addition to directly killing tumor cells by PDT. Therefore, this nanoplatform may address the critical problems of poor therapeutic effects of conventional PDT and tumor immune resistance due to tumor environmental characteristics (e.g., tumor hypoxia, excessive H^+^ ions, and immune resistance) and offer an effective strategy for synergistic PDT and immunotherapy against malignant tumors. Additionally, single‐stranded DNA molecules can also be used as building blocks in photoresponsive biohybrids. In one case, Yang et al. first developed a novel DNA nanostructure via the coordination between AS1411 DNA G quadruplexes and calcium ions and then utilized the nanoscale coordination polymers to incorporate hemin and Ce6 into the G‐quadruplex structure (Figure 3D).^106^ The obtained nanostructure enabled the ROS generation of Ce6 inside the cell nuclei, along with catalase‐mimicking DNAzyme functions of the hemin and G‐quadruplexes, thereby greatly enhancing PDT efficacy in vivo.

#### 3.3.2 Cell membrane‐based photoresponsive biohybrids

**FIGURE 4:**
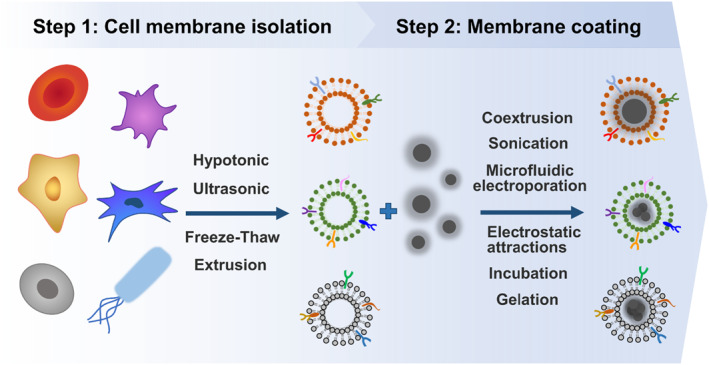
Schematic illustrating the fabrication methods of cell membrane‐coated biohybrids.

Cell membranes are thin semipermeable membranes composed of various biomolecules, including proteins, lipids, and carbohydrates.^109^ The carbohydrates and transmembrane or membrane‐anchored proteins are mainly involved in cell interfacing functionalities, while the bilayer lipid structure creates a semi‐permeable barrier toward the external environment.^110^ Cell membrane isolation was first achieved by Michael L.S. and colleagues in 1976 by preparing erythrocyte membranes through hypotonic lysis and isotonic resealing treatment.^111^ In 2011, the Zhang group first reported the cell membrane coating technology using natural erythrocyte membranes to coat polymeric nanoparticles.^112^ The resultant membrane‐coated nanoparticle faithfully preserved the bilayer structures and functional surface proteins of the erythrocyte membranes, which enabled the biomimetic nanoparticle to exhibit the long circulation property from the source cells. Besides erythrocyte membranes, a variety of natural membrane‐coated particles have been developed with different functions for biomedical applications.^24^, ^25^, ^26^, ^113^, ^114^, ^115^, ^116^, ^117^, ^118^ For example, macrophage cell membrane‐cloaked systems were demonstrated for targeted delivery of drugs to inflammatory sites and tumorous tissues^119^; cancer cell membrane‐coated particles could enhance cellular uptake, tumor‐specific targeting, and immune response^48^, ^49^, ^50^, ^51^, ^52^, ^53^; and bacterial cell membrane‐coated particles also exhibited tumor‐targeting and immune stimulation features in O_2_‐dependent cancer treatment.^54^, ^55^ The fabrication process of the membrane‐coated particles mainly includes two steps (Figure 4): (i) cell membranes are first isolated via hypotonic treatment, ultrasonic cell disruption or repeated freezing and thawing; and (ii) the particle cores are then coated by membranes through mechanical coextrusion, sonication, microfluidic electroporation, or electrostatic attractions, and so on.^110^, ^113^, ^118^, ^120^, ^121^, ^122^

More recently, cell membranes have been employed as effective design strategies for biomimetic photoresponsive materials in tumor phototherapy.^6^, ^64^, ^67^, ^68^, ^69^, ^123^, ^124^, ^125^, ^126^, ^127^, ^128^, ^129^, ^130^ For example, Ding et al. prepared biomimetic nanovesicles by camouflaging hemoglobin‐linked semiconducting polymer nanoparticles with RBC membranes.^69^ Their results demonstrated that membrane coating enabled the long‐term circulation of inner nanoparticles, improved their photostability and accumulation in tumors, and eventually enhanced the synergetic chemo‐PDT efficacy against hypoxic tumors. Similarly, Yang et al. coated the aggregation‐induced emission nanodots with mature dendritic cell membranes, which rendered the biomimetic biohybrids with a hitchhiking function for T cell‐mediated cancer targeting and enhanced PTT.^124^ Moreover, cytomembranes of hybrid cells, which retained the membrane proteins of different cell lines, have been used for functionalization of PS for combined tumor therapy.^49^, ^131^ Liu et al. proposed a tumor‐specific immunotherapy‐based nanoplatform by cloaking PS‐containing metal‐organic frameworks with the cytomembranes derived from dendritic cells and parent cancer cells.^131^ Benefiting from the whole cancer antigens and immunological co‐stimulatory molecules, the cytomembrane‐based biohybrid exhibited ultrahigh immunotherapeutic effects against tumors compared to PDT efficacy.

#### 3.3.3 Eukaryotic cell‐based photoresponsive biohybrids

**FIGURE 5:**
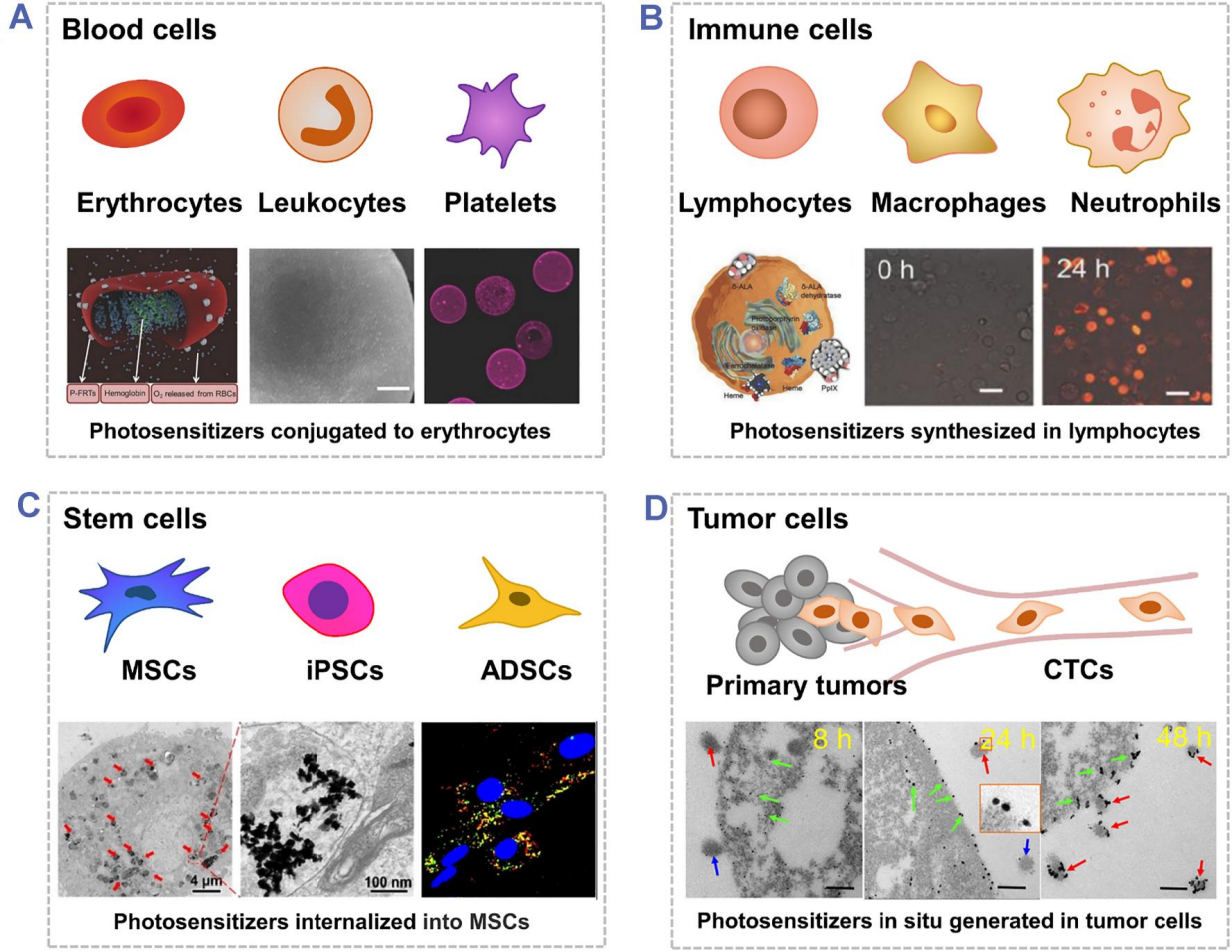
Representative photoresponsive biohybrids by incorporating various living eukaryotic cells, including (A) blood cells, Reproduced with permission.^96^ Copyright 2016, John Wiley and Sons. (B) immune cells, Reproduced with permission.^91^ Copyright 2018, John Wiley and Sons. (C) stem cells, Reproduced with permission.^79^ Copyright 2022, American Chemical Society, and (D) tumor cells. Reproduced with permission.^92^ Copyright 2019, American Chemical Society.

A variety of intact eukaryotic cells, including blood cells (e.g., erythrocytes,^28^, ^56^, ^96^ leukocytes, and platelets^83^), immune cells (e.g., macrophages,^57^, ^58^, ^78^, ^132^, ^133^ lymphocytes,^91^ and neutrophils^134^), stem cells (e.g., mesenchymal stem cells [MSCs]),^73^, ^76^, ^77^, ^79^, ^86^, ^135^, ^136^, ^137^ induced pluripotent stem cells,^75^, ^138^ adipose‐derived stem cells,^74^ neural stem cells,^85^ and tumor cells^92^ have also been used to engineer photoresponsive biohybrids for tumor therapy (Figure 5).

RBCs (i.e., erythrocytes) are the most abundant cells in our body (over 80% of all cells) and have been widely used as drug delivery vehicles for over 30 years.^28^, ^139^, ^140^ Benefiting from their prolonged circulation time and O_2_ transport function, RBCs were used as carriers for the combined delivery of O_2_ and PS in PDT (Figure 5A).^96^, ^141^ Phototherapy has also been combined with immunotherapy by using photoresponsive immune cells.^142^ In one case, Zheng et al. used tumor‐targeted photoresponsive lymphocytes modified with δ‐aminolevulinic acid to generate vesicle‐like apoptotic bodies and synthesize anti‐neoplastic drugs (protoporphyrin X, PpIX) for systematic cancer therapy (Figure 5B).^91^

Apart from the tumor‐tropic immune cells, stem cells also preferentially migrate to tumor regions because of the interactions between the chemokines released from tumor tissues and the chemokine receptors expressed on the surfaces of stem cells, which can increase the tumor‐targeting efficiency of therapeutic agents in stem cell‐mediated delivery systems.^74^, ^79^, ^86^, ^137^, ^143^ For example, Ning et al. developed photosensitive MSCs loaded with mesoporous silica‐coated gold nanostars integrated with ICG as the theranostic platform for the spatiotemporal tracking of MSCs and imaging‐guided PTT in treating breast cancers (Figure 5C).^79^ In this biohybrid platform, the gold nanostars were coated with a mesoporous silica shell for better photostability, which also provided large‐surface areas for the subsequent integration of ICG molecules. MSCs acted as the carriers to improve the intratumoral distribution and retention of therapeutic agents, while the photoresponsive hybrid nanoparticles enabled the real‐time tracking of MSCs with a high spatiotemporal resolution.

In addition, PS could be synthesized in situ within tumor cells and then exocytosed as nanoparticle‐trapped vesicles with retained tumor antigens for combinatorial photo‐immunotherapy (Figure 5D).^92^ The tumor‐derived vesicles were further internalized by dendritic cells and secreted as dendritic cell‐derived vesicles, which could not only improve the biocompatibility and immunological property of nanoparticles, but also minimize the possibility of metastasis induced by tumor‐derived vesicles.

Both the whole eukaryotic cells and their membranes have been widely employed to develop biohybrid flatforms in tumor therapy. Compared with the synthetic materials, they are endogenic and are considered as much more biocompatible with multiple biofunctions originated from the parent cells.^144^ For the cell membrane‐coating nanoparticles, the retention of the membrane proteins makes the nanoparticles more feasible to prolong the circulation time, improve the drug accumulation in tumor tissues, and thus enhance the therapeutic efficiency for treating cancers.^68^, ^109^, ^122^ While for whole erythrocyte cell‐based biohybrids, the living vehicle cells are used to encapsulate or bind therapeutic agents and can be preferentially recruited and accumulated into the inflammatory tumor tissues.^41^, ^144^, ^145^

#### 3.3.4 Bacteria‐based photoresponsive biohybrids

Bacteria play central roles in energy metabolism and generate important biomolecules and substrates for mammalian organisms.^146^ Bacteria have been exploited in various areas of biomedical sciences and biotechnology, including bioenergy, pharmaceuticals, and bioremediation.^147^, ^148^, ^149^, ^150^ Several species of bacteria, including Gram‐positive anaerobes (e.g., *Clostridium beijerinckii* and *Bifidobacterium bifidum*) and Gram‐negative facultative anaerobes (e.g., *Salmonella typhimurium*), have been found to specifically colonize in tumor areas.^36^, ^40^, ^139^, ^151^ Such specificity can be exploited for localized drug delivery to specific tumor areas.^71^, ^89^, ^97^, ^152^ Bacteria‐based biohybrid systems also exhibit tumor‐targeting functions and improved performance for either single or synergistic tumor therapy.^2^, ^39^, ^128^, ^148^, ^152^, ^153^, ^154^, ^155^

**FIGURE 6:**
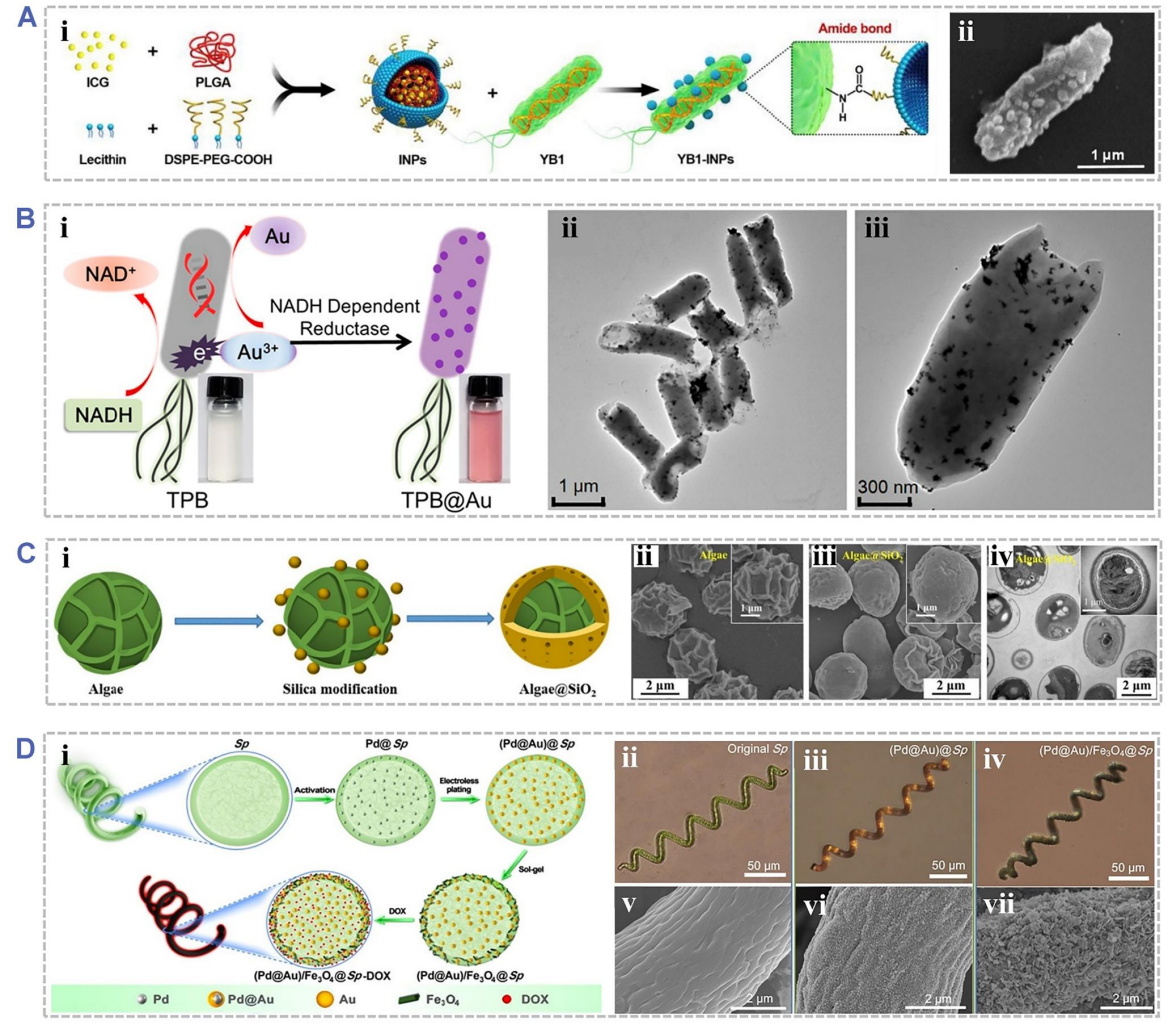
Design of various microorganism‐based photoresponsive biohybrids. (A) Design of the nanophotosensitizer‐engineered Salmonella bacteria (YB1‐INPs): (i) Schematic illustrating that *Salmonella typhimurium* YB1 were covalently attached by the indocyanine green (ICG)‐loaded nanoparticles (ii) SEM images of YB1‐INPs. Reproduced with permission.^38^ Copyright 2019, Elsevier. (B) Design of a thermally sensitive programmable therapeutic system using bacteria *Escherichia coli* MG1655 decorated with biomineralized photothermic gold nanoparticles (TPB@Au): (i) Biosynthesis mechanism of AuNPs by thermally sensitive programmable bacteria (TPB) through enzymatic reduction; (ii, iii) TEM images of TPB@Au. Reproduced with permission.^59^ Copyright 2018, American Chemical Society. (C) Design of biomineralized biohybrid photosynthetic algae: (i) Schematic illustrating the biohybrid algae (Algae@SiO_2_) synthesized by a one‐step biomimetic silicification method; (ii, iii) SEM images of algae and Algae@SiO_2_; (iv) TEM images of Algae@SiO_2_. Reproduced with permission.^95^ Copyright 2020, American Chemical Society. (D) Design of the magnetic microrobots based on *Spirulina* templates: (i) Fabrication processes of (Pd@Au)/Fe_3_O_4_@*Sp.*‐DOX microrobots; (ii–iv) Optical and (v–vii) SEM images of original *Sp.* (ii, v), (Pd@Au)@*Sp.* (iii, vi), and (Pd@Au)/Fe_3_O_4_@*Sp.* (iv, vii). Reproduced with permission.^31^ Copyright 2019, American Chemical Society.

To date, bacteria‐based photoresponsive biohybrids have attracted great attention in phototherapy due to the good bioactivity, high drug loading efficacy, and tumor targeting capacity of the bacterial components.^2^, ^5^, ^29^, ^30^, ^38^, ^39^, ^59^, ^70^, ^71^, ^80^, ^84^, ^97^, ^156^, ^157^ For example, a biotic/abiotic cross‐linked biohybrid system was developed with photosensitive ICG‐loaded nanoparticles covalently attached to the surface of a genetically modified *S. typhimurium* strain (Figure 6A).^38^ Benefiting from the hypoxia‐targeting ability, the biohybrid system exhibited excellent PTT efficacy to kill tumor cells while avoiding damage to normal tissues, indicating a bacteria‐mediated therapeutic strategy for the treatment of deep‐seated or large solid tumors. In another case, Fan et al. reported a bacteria‐mediated therapeutic system using *Escherichia coli* MG1655 as a vehicle to deliver TNF‐α plasmids and Au nanoparticles to tumor regions (Figure 6B).^59^ The AuNPs and TNF‐α plasmids were orally delivered and transported into internal microcirculation via transcytosis and then accumulated at tumor sites. Importantly, the photothermal heat generated from AuNPs could remotely activate TNF‐α expression to kill tumor cells. This study suggested that bacteria‐based photoresponsive biohybrids could be an effective strategy to overcome the barrier in current oral administration for tumor therapy and achieve satisfactory tumor‐targeting therapeutic outcomes.

#### 3.3.5 Algae‐based photoresponsive biohybrids

Microalgae are important photosynthesizers that largely contribute to the O_2_ budget on Earth.^158^, ^159^, ^160^ Recently, there has been a strong interested in using algae for biotechnological and biomedical applications, including tumor therapy.^37^, ^81^, ^158^, ^161^, ^162^ Often, the algae‐based biomaterials are used as light‐driven O_2_ generators and carriers, allowing for a sustainable delivery of O_2_ to lesion sites.^34^, ^35^, ^62^, ^65^, ^149^, ^163^, ^164^, ^165^, ^166^ Additionally, hybrid photosynthetic biomaterials have been fabricated via 3D bioprinting for efficient algal cultivation in algal biotechnology and for environmental applications (e.g. coral restoration).^167^, ^168^ Such hybrid living photosynthetic biomaterials could find wide applications in biomedical engineering and environmental applications.

For the treatment of cancers, algae‐based photoresponsive biohybrids are developed for phototherapy mainly because they can provide sustainable O_2_ under light irradiation and thereby alleviate the hypoxic microenvironment in tumor tissues.^31^, ^32^, ^60^, ^61^, ^66^, ^72^, ^94^, ^163^, ^164^, ^169^, ^170^, ^171^, ^172^, ^173^, ^174^ For example, Wang et al. reported a biomineralized biohybrid algae (Algae@SiO_2_) for alleviating tumor hypoxia and synergistic radio PDT (Figure 6C).^174^ The algae *Chlorella vulgaris* was modified with silica via a biomimetic silicification method. The silica shell separated the algae from the external environment, reducing their cytotoxicity while the photosynthetic activities are retained for O_2_ production. Thus, the biohybrid algae could function as an effective O_2_‐evolving photodynamic system to relieve the tumor hypoxia and thus improve the PDT efficacy for tumor therapy.

It is worth mentioning that the intrinsic morphology of several algae cells, for example, the helical structures of *Spirulina* (*Sp.*), could be for ideal structural templates for fabricating biohybrid materials. In one case, helical microswimmers were fabricated by coating Fe_3_O_4_ nanoparticles onto the *Sp.* microalgae surfaces while preserving their structural features and intrinsic functionalities.^31^, ^175^ Benefiting from both the biological organic matter and the integrated magnetic component, the obtained magnetic biohybrids exhibited intrinsic autofluorescence, magnetic resonance signals, and tunable biodegradability, indicating a high potential for imaging‐guided therapy. In another case, Wang et al. presented a facile method for mass production of magnetic microrobots by using the *Sp.* cells as structural templates (Figure 6D).^31^ In this route, (Pd@Au)@*Sp* was first obtained by synthesizing the core‐shell‐structured Pd@Au nanoparticles in *Sp.* templates for PTT. Subsequently, (Pd@Au)/Fe_3_O_4_@*Sp* was fabricated by depositing Fe_3_O_4_ nanoparticles on as‐(Pd@Au)@*Sp* for magnetic responsive ability. Moreover, chemotherapeutic doxorubicin (DOX) is further loaded to establish the (Pd@Au)/Fe_3_O_4_@*Sp*.‐DOX platform for additional chemotherapeutic efficacy. Therefore, such biohybrids not only possessed targeted‐delivery efficacy via magnetic propulsion, but also exhibited enhanced synergistic chemo‐PTT capacity. This study indicated a promising and efficient algae‐based responsive platform for targeted delivery, drug loading, and synergistic tumor therapy.

## 4 TUMOR THERAPEUTIC MODALITIES WITH ENGINEERED PHOTORESPONSIVE BIOHYBRIDS

### 4.1 Tumor PTT

In traditional PTT, tumor therapy is often facilitated by photoresponsive agents like gold nanoparticles that trigger the physical hyperthermia to ablate tumor cells.^33^, ^62^, ^122^, ^124^ Unfortunately, traditional photoresponsive agents have inherent shortcomings, including poor biocompatibility, low tumor targeting efficiency, short blood circulation time, and intrinsic tumor heat endurance.^50^, ^76^, ^93^, ^108^, ^176^ Recently, many attempts have been made to develop engineered photoresponsive biohybrids for overcoming these limitations and improving tumor therapeutic outcomes.^30^, ^32^, ^36^, ^38^, ^59^, ^68^, ^83^, ^84^, ^169^, ^177^, ^178^

**FIGURE 7:**
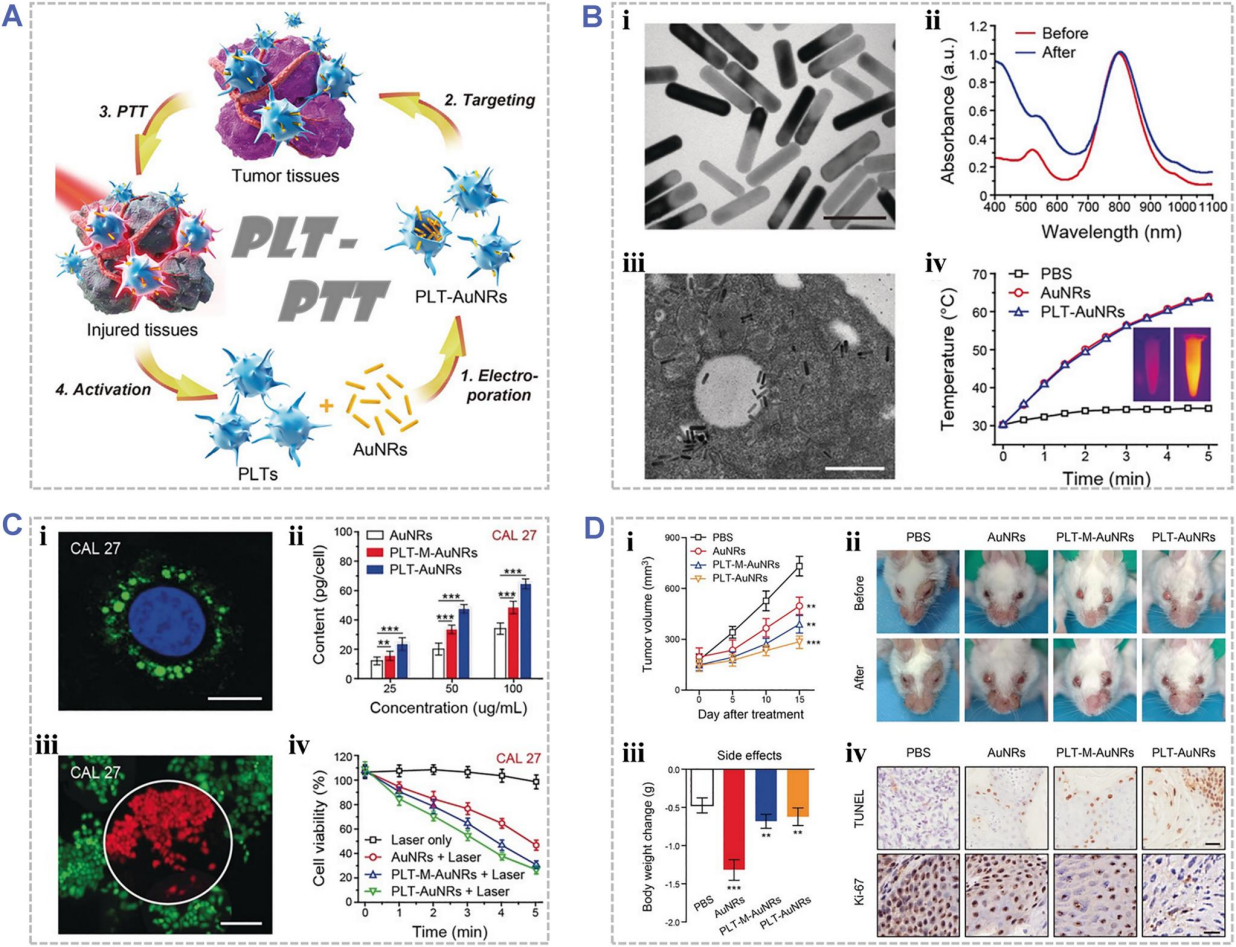
Engineered photoresponsive biohybrids for photothermal therapy. (A) Schematic illustrating the design and construction of platelet‐facilitated photothermal tumor therapy (PLT‐PTT). (B) Physicochemical characterization of PLT‐AuNRs: (i) Representative TEM image of AuNRs. Scale bar, 50 nm; (ii) UV/Vis‐NIR absorption spectrum of AuNRs before and after surface modification of BSA; (iii) Representative TEM image of PLT‐AuNRs. Scale bar, 250 nm; (iv) Temperature curves of PBS and PBS containing AuNRs or PLT‐AuNRs under 808 nm laser irradiation. The inset shows IR thermal images of PLT‐AuNRs before (left) and after (right) the laser irradiation for 5 min. (C) In‐vitro characterization of immune evasion and PTT effect of PLT‐AuNRs: (i) Representative confocal image of singe CAL 27 cancer cell after incubation with PLT‐AuNRs. Scale bar, 10 μm; (ii) Nanoparticle uptake by CAL 27 cells at different incubated concentrations; (iii) Representative confocal image of CAL 27 cells after the laser irradiation. Scale bar, 100 μm. White circles indicate the laser irradiation sites; (iv) Viability of CAL 27 cancer cells after various treatments for different durations. (D) In‐vivo photothermal anticancer effect evaluation: (i) Tumor volume curves after various treatments; (ii) Representative photos of HNSCC‐bearing mice before and after various treatments; (iii) Treatment side effects were assessed by mice body weight in each group; (iv) Representative TUNEL‐ and Ki‐67‐stained tumor slice images of mice after various treatments. Scale bars, 50 and 25 μm in TUNEL‐ and Ki‐67‐stained slices, respectively. Compared with the PBS group, ** and *** individually indicates *p* 〈 0.01 and *p* 〈 0.001. Reproduced with permission.^83^ Copyright 2018, John Wiley and Sons.

A typical case was presented with a platelet‐facilitated PTT (PLT‐PTT) strategy by Rao et al., in which they used the PLTs as carriers loading PTAs for targeted delivery and enhanced the PTT effect in tumor tissues (Figure 7A).^83^ Gold nanorods (AuNRs) of about 50 nm in length and 12 nm in diameter were first synthesized and then loaded into PLTs via electroporation to obtain the AuNR‐loaded PLTs (PLT‐AuNRs), which preserved a good photothermal property of AuNRs (Figure 7B). More importantly, PLT‐AuNRs inherited the capabilities to evade phagocytosis and target to cancer cells from PLTs. The nanoparticles could be uptaken by the cancer cells, allowing for the selectively tumor killing efficacy under NIR irradiation (Figure 7C). The in vivo PLT‐PTT effect was demonstrated to effectively inhibit the growth of squamous cell carcinoma in mice (Figure 7D), indicating unique self‐reinforcing characteristic of eukaryotic cell‐based photosensitive biohybrids in cancer therapy. In another case, Hosseini et al. reported a gold helix phototheranostic biohybrid for image‐guided targeted PTT in breast cancer.^32^ They used *Spirulina platensis* (SP) microalga as helix structural templates for the fabrication of helical architecture of Au nanoparticles and improvement of the biosafety for clinical applications. The Au‐SP biohybrid was prepared with quasi‐spherical Au nanoparticles embedded throughout the SP cells. The high X‐ray absorbance of AuNPs and autofluorescence of SP cells were employed for dual‐modal computed tomography and fluorescence imaging. In addition, the high tumor inhibition effects of Au‐SP biohybrids were demonstrated both in vitro and in vivo, which indicating a promising strategy to utilize microorganism‐based photoresponsive biohybrids as a theragnostic system for tumor therapy.

### 4.2 Tumor PDT

Similar to PTT, PDT has been regarded as a promising approach for tumor therapy.^6^, ^12^, ^52^, ^73^, ^179^ Unlike PTT, PDT uses PS to absorb light energy to generate cytotoxic ROS, for example, singlet oxygen (^1^O_2_), superoxide (O_2_˙^−^), hydroxyl radicals (·OH), and hydrogen peroxide (H_2_O_2_), that can induce the apoptosis and/or necrosis of tumor cells.^88^, ^106^, ^129^, ^165^, ^180^, ^181^ Current PS mainly includes organic porphycenes, small‐molecule dyes, gold nanoclusters, metallic nanoparticles, two‐dimensional materials, and quantum dots.^173^, ^182^ Despite the significant progress in PDT, the ROS production efficiency mainly suffers from (i) shortage of O_2_ supply in deep‐seated tumors and (ii) limited penetration depth of visible light sources.^29^, ^66^, ^107^, ^151^, ^170^, ^171^, ^183^ To address the two issues, many strategies have been proposed including the development of engineered photoresponsive biohybrids.^38^, ^60^, ^61^, ^72^, ^127^, ^164^, ^172^

**FIGURE 8:**
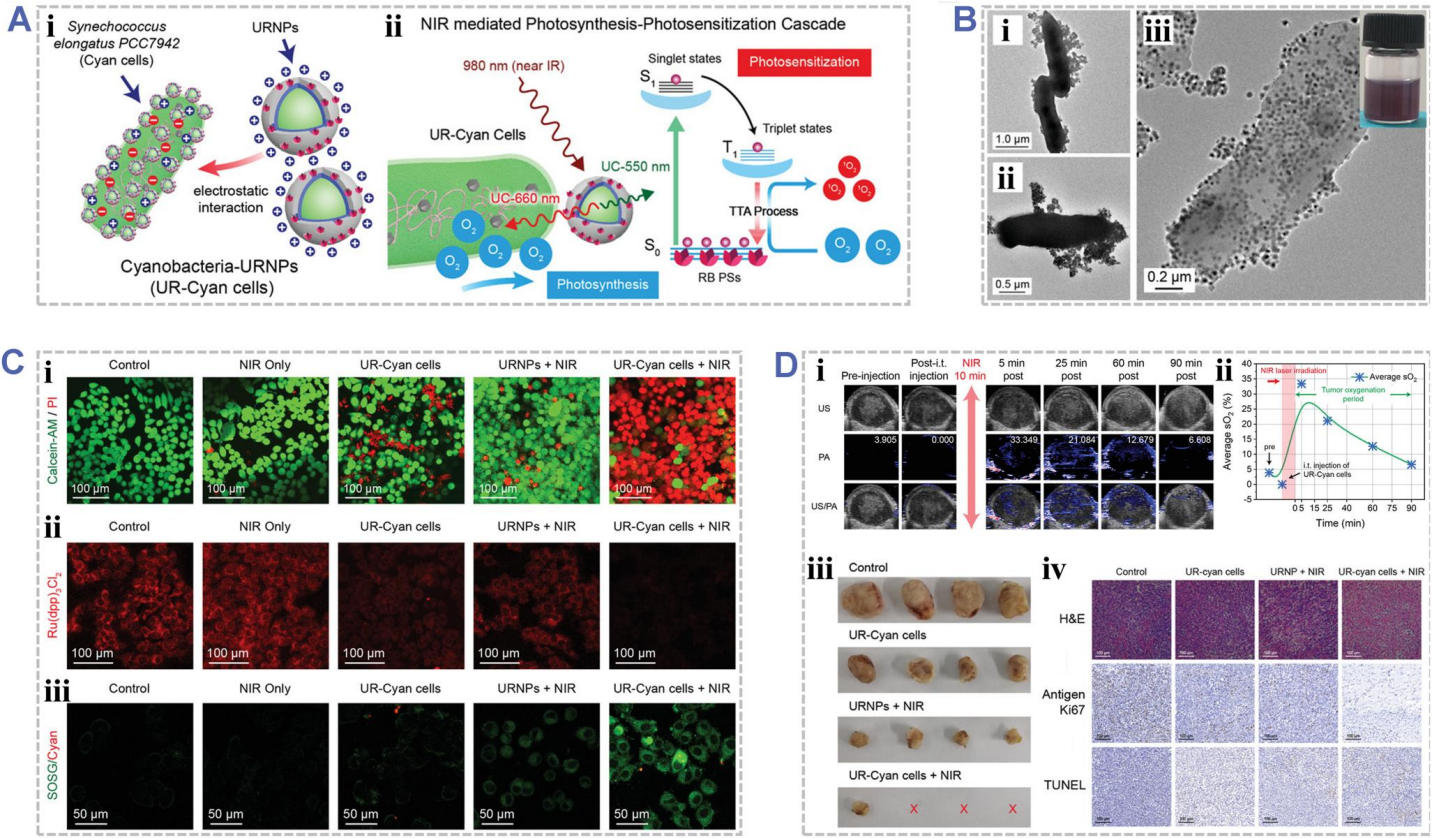
Engineered photoresponsive biohybrids for photodynamic therapy. (A) Schematic illustrating the design and construction of upconversion nanoparticles (URNPs) hybridized cyanobacterial cells for near‐infrared‐mediated photosynthesis and enhanced photodynamic therapy: (i) hybridization of cyanobacterial cells with through electrostatic interaction; (ii) IR‐laser induced and upconversion‐mediated photosynthetic and photosensitization cascade. (B) Morphology characterizations of the hybridized UR‐Cyan cells: (i, ii) Bio‐TEM images of UR‐Cyan cells; (iii) TEM image of UR‐Cyan cells. Inset, a digital photograph of 1 mL solution containing UR‐Cyan cells. (C) Confocal images of (i) Calcein‐AM/PI dual‐stained, (ii) Ru(dpp)3Cl2 stained, and (iii) SOSG stained 4T1 tumor cells after various treatments, validating augmented cellular PDT performance by the upconversion‐mediated photosynthesis and photosensitization cascade. (D) In‐vivo photosynthetic‐photosensitization cascade and animal PDT performance. (i) Time‐course ultrasound and photoacoustic images of tumor xenografts of mice injected with UR‐Cyan cells and under NIR laser irradiation; (ii) Time‐course average O_2_ levels derived from the PA images; (iii) Digital photographs of the dissected tumors from mice in different treatment groups; (iv) Microscopic images of H&E immunostained, antigen Ki67‐stained, and TUNEL‐stained tumor sections from different treatment groups after the PDT. Reproduced with permission.^72^ Copyright 2021, John Wiley and Sons.

For example, Shi group reported an NIR‐driven PDT platform (named as UR‐Cyan cells) by hybridizing the PS rose bengal (RB)‐loaded upconversion nanoparticles (UCNPs) with photosynthetic cyanobacterial cells (Figure 8A).^72^ In this platform, the UCNPs could effectively upconvert the NIR laser into visible lights. The generated visible lights not only facilitated photosynthesis by cyanobacterial cells, but also activated RB to react with surrounding O_2_ to produce ^1^O_2_. The UR‐Cyan cells were prepared via conjugation of UCNPs onto the surface of the cyanobacterial cells (Figure 8B). Under NIR laser irradiation, the majority of tumor cells were killed by PDT with UR‐Cyan cells (Figure 8C). After intratumoral injection into tumor xenograft‐bearing mice, the UR‐Cyan cells could effectively relieve the tumor hypoxia and inhibit the tumor growth under NIR irradiation (Figure 8D). This design offers a practical strategy to augment PDT therapeutic effects with high tissue penetration and self‐supply of O_2_. In another example, Zhang group decorated UCNPs with thylakoid membranes of chloroplasts to realizing O_2_ self‐supply and simultaneous ROS production for hypoxic tumor therapy.^127^ In this unique photosystem, the UCNPs can emit the red light upon 980 nm laser irradiation, which activated the PDT system derived from chloroplasts, facilitated O_2_ generation and ROS production, and effectively eradicated the hypoxic tumors in mice. This study provides a new PDT strategy for hypoxic tumor therapy based on engineered photoresponsive biohybrids.

### 4.3 Synergistic tumor therapy

Despite the significant progress of phototherapy in tumor treatment, significant limitations remain, including limited light penetration depth and low therapeutic efficacy hindered by local hypoxic microenvironments at tumor sites.^65^, ^69^, ^87^, ^154^, ^172^ Specifically, absorption of visible and NIR radiation by human tissues limit phototherapy to superficial tissues, leaving deep‐seated tumors or metastatic tumors untreated. Additionally, local tumor hypoxia has been shown to promote cancer cell rapid proliferation and induce drug resistance, which further increases the difficulty in cancer treatment.^34^, ^53^, ^106^, ^131^ Recently, engineered photo responsive biohybrids have been engineered to combine different antitumor therapeutic strategies for treating hypoxic tumors, such as synergistic PTT, PDT, chemotherapy, radiotherapy, and/or immunotherapy.^13^, ^31^, ^34^, ^35^, ^51^, ^64^, ^78^, ^80^, ^89^, ^90^, ^97^, ^103^, ^121^, ^128^, ^130^

**FIGURE 9:**
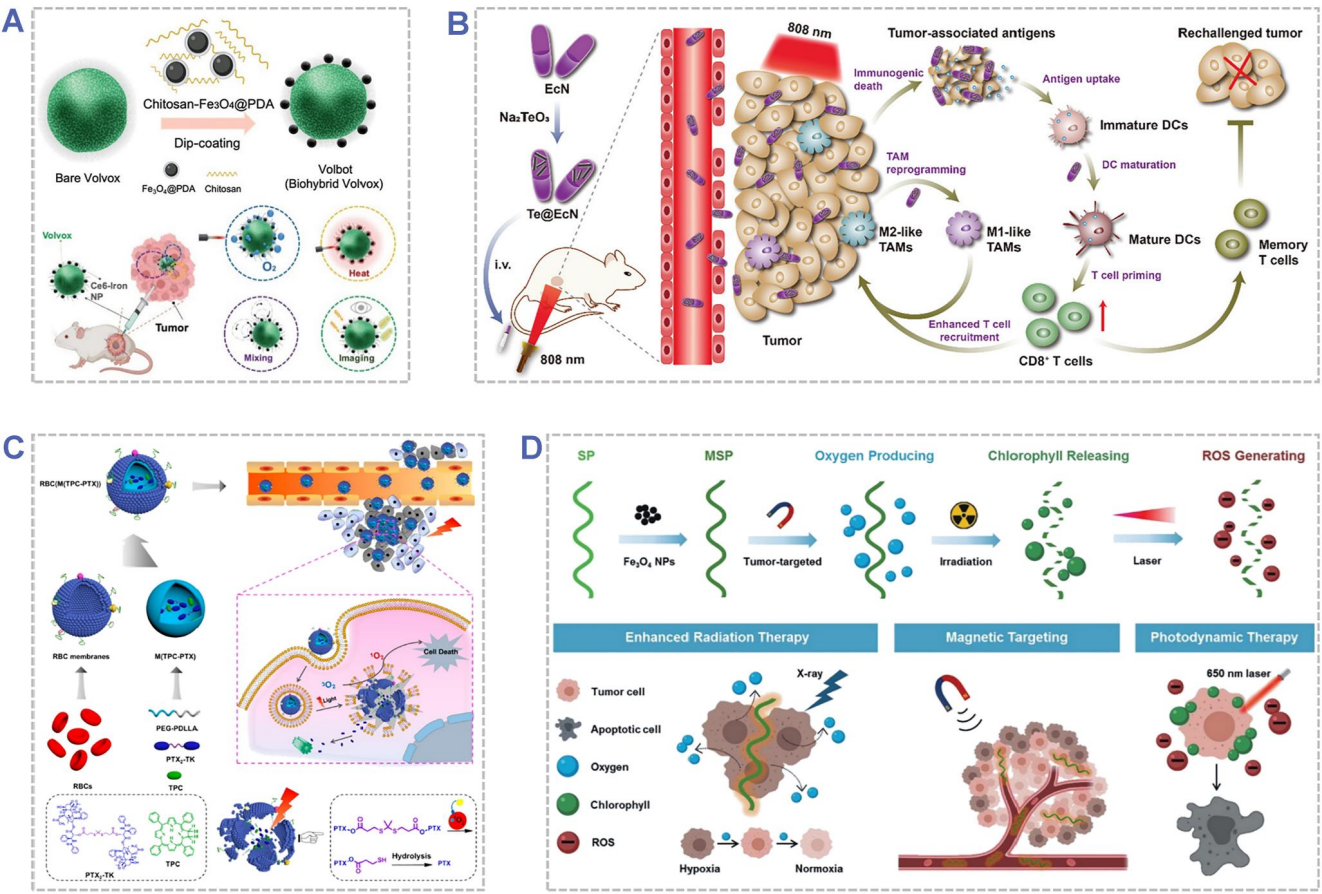
Engineered photoresponsive biohybrids for synergistic tumor therapy. (A) Schematic showing the design of volvox microalgae‐based biohybrid microrobotics for image‐guided photodynamic/photothermal combination cancer therapy. Reproduced with permission.^34^ Copyright 2022, John Wiley and Sons. (B) Schematic showing the design of the biohybrid therapeutic platform (Te@EcN) for photothermal‐immunotherapy against advanced malignant tumors. Reproduced with permission.^97^ Copyright 2022, John Wiley and Sons. (C) Schematic showing the design of red blood cell membrane‐camouflaged biohybrid prodrug nanoparticles for light‐triggered on‐demand drug release and combined photodynamic/chemotherapy. Reproduced with permission.^130^ Copyright 2018, American Chemical Society. (D) Schematic showing the design of the photosynthetic biohybrid nanoswimmer system to alleviate tumor hypoxia for FL/PA/MR imaging‐guided enhanced radio‐photodynamic synergetic therapy. Reproduced with permission.^35^ Copyright 2020, John Wiley and Sons.

For instance, Wang et al. developed an all‐in‐one volvox‐based biohybrid microrobot (denoted as Volbot) for photosynthesis‐promoted synergistic PTT/PDT (Figure 9A).^34^ This biohybrid microrobot was prepared by integrating a green algae (*volvox*), photodynamic agents (Ce6), and PTAs (polydopamine functionalized magnetic nanoparticles, Fe_3_O_4_@PDA) into one system. The photosynthetic algae could generate O_2_ to alleviate tumor hypoxia, and photosynthesis simultaneously promoted the PDT effect of Ce6 under red light and PTT effect of Fe_3_O_4_@PDA under NIR light. In another case, Yao et al. reported a bacteria‐based biohybrid therapeutic platform with tumor targeting ability for synergistic PTT and immunotherapy (Figure 9B).^97^ In this platform, the tellurium nanorods (TeNRs) were synthesized inside the *E. coli* Nissle 1917 (EcN) cells via facile intracellular biosynthesis. Owing to the high photothermal conversion efficiency of TeNRs along with co‐stimulation by probiotic EcN as immunoadjuvants, the PTT efficacy was boosted in the immunosuppressive tumor environment, which could effectively eliminate advanced malignant tumors, prevent tumor metastasis and recurrence, and prolong survival in tumor‐bearing mice.

In clinic, chemotherapy uses chemical drugs to kill widespread or metastatic tumor cells, while its efficacy is severely limited by the side effects caused by the non‐specificity and poor‐selectivity to cancerous cells.^6^, ^126^, ^130^, ^157^ Although radiotherapy uses high‐energy radiation to destroy tumor cells in confined areas, it has long‐term side effects, and tumor resistance may occur because of the insufficient O_2_ supply in hypoxic tumors.^35^, ^94^, ^163^, ^174^ In this regard, engineered photoresponsive biohybrids offer opportunities to design synergistic tumor therapeutic platforms with enhanced biosafety and antitumor performance.^169^, ^184^ For example, Pei et al. designed RBC membranes‐camouflaged dimeric prodrug nanoparticles (RBC(M(TPC‐PTX))) for synergistic PDT and chemotherapy (Figure 9C).^130^ In this platform, the inner 5,10,15,20‐tetraphenylchlorin (TPC, a PS) and paclitaxel (PTX, a chemical drug) dimer‐loaded nanoparticle core was responsible for light‐triggered ROS generation for PDT and on‐demand PTX release for chemotherapy, while the outer RBC membrane shell could overcome the rapid blood clearance and improve tumor accumulation, which enhanced the therapeutic efficacy and decreased the toxicity of each therapeutic agent. In another example, Zhong et al. utilized the superparamagnetic‐Fe_3_O_4_‐functionalized *S. platensis* as photosynthetic biohybrid nanoswimmer systems for tumor‐targeted imaging and enhanced synergistic PDT/radiotherapy in hypoxic solid tumors (Figure 9D).^35^ The living *S. platensis* not only acted as a PS due to its ROS generation ability for PDT, but also utilized as a theragnostic agent owing to the native chlorophyll for fluorescence and photoacoustic imaging. In addition, Fe_3_O_4_ nanoparticles could magnetically target to the tumor site and functions as contrast agents for magnetic resonance imaging. More importantly, the living biohybrid system could generate O_2_ in situ to relieve tumor hypoxia, thus enhancing the radiotherapy efficacy. This study provide a promising therapeutic platform for tumor‐targeted imaging and synergistic therapy.

Taking together, all above examples suggest that the integration of synthetic and biological components enables multifunctionality that cannot be achieved in one single system. These versatile synergistic therapeutic modalities based on engineered photoresponsive biohybrids can offer innovative strategies for enhanced therapeutic performances by modulating the tumor microenvironment.

### 4.4 Tumor therapy combined with tissue regeneration

Current clinical treatment strategies of solid tumors are surgical resection, chemotherapy, and radiotherapy.^3^, ^4^ However, these treatment modalities have formidable obstacles such as the critical tissue defects after the surgical resection, undesirable chemotherapy resistance and severe side effects, and low therapeutic efficacy.^5^, ^6^, ^7^, ^8^ Hence, it is necessary to develop a more efficient strategy to achieve both tumor therapy and tissue regeneration.^185^, ^186^, ^187^, ^188^, ^189^

**FIGURE 10:**
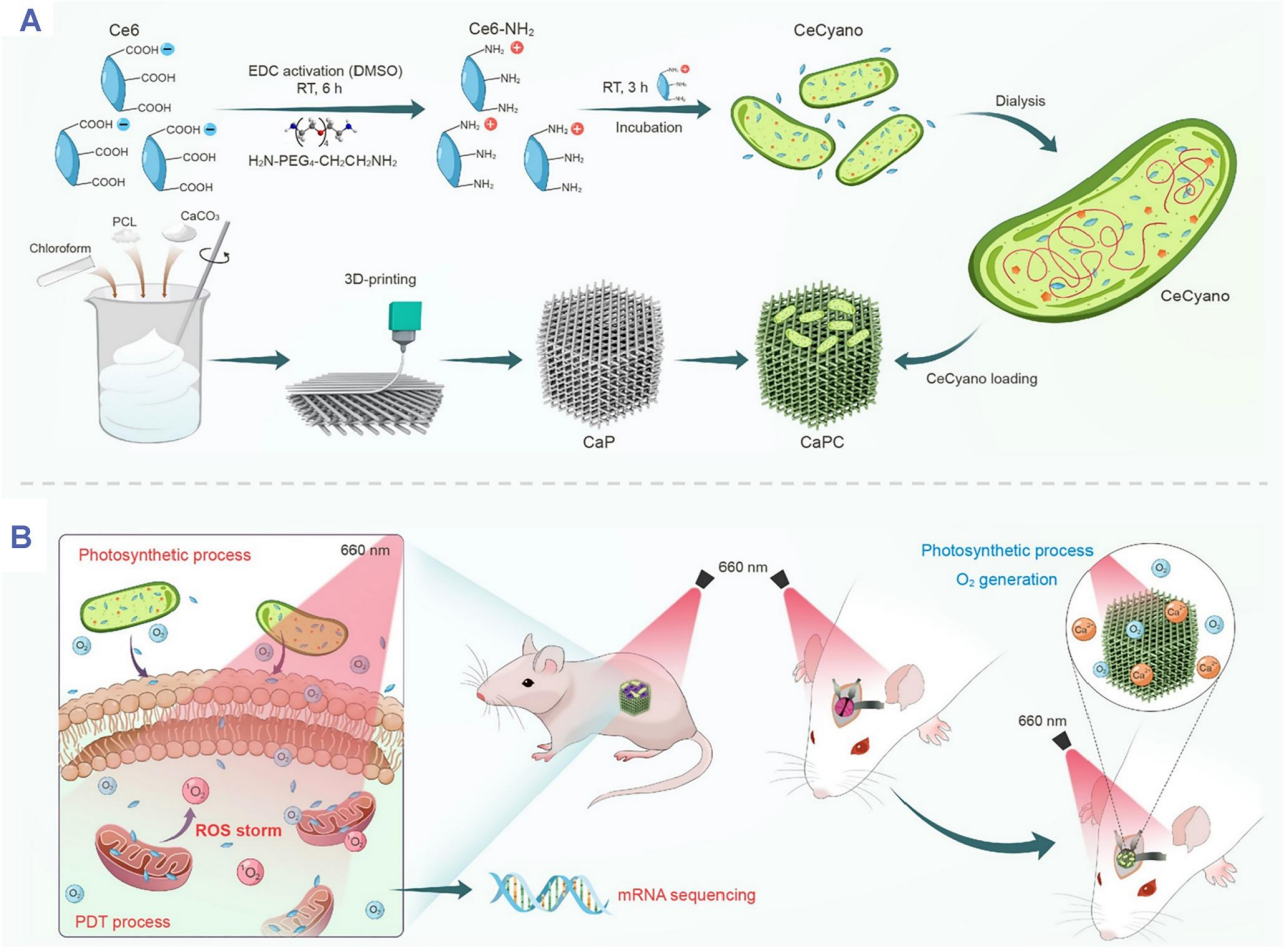
Engineered photoresponsive biohybrids for tumor therapy combined with tissue regeneration. (A) Schematic showing the preparation of the photosensitizer‐engineered biohybrid scaffolds by integrating the photosensitive and photosynthetic Ce6‐contained cyanobacteria onto 3D‐printed CaCO_3_‐PCL scaffolds. (B) Under 660 nm laser exposure, the engineered scaffolds could generate O_2_ by photosynthesis and subsequently activate Ce6, leading to the production of reactive oxygen species (especially the singlet oxygen) for high‐effective PDT against osteosarcoma. Meanwhile, the excessively generated O_2_ and CaCO_3_ components from the scaffolds could also prompt bone regeneration after the elimination of osteosarcoma. Reproduced with permission.^82^ Copyright 2021, Elsevier.

Engineered photoresponsive biohybrids have emerged as promising therapeutic platforms by combining the tumor phototherapy with tissue regeneration together. For instance, He et al. developed a multifunctional photosynthetic O_2_‐self‐generated therapeutic platform by integrating the photosynthetic Ce6‐contained cyanobacteria onto 3D‐printing CaCO_3_‐PCL scaffolds for enhanced PDT against osteosarcoma and simultaneous bone regeneration (Figure 10).^82^ The preparation process of the biohybrid scaffold could be divided into three steps (Figure 10A): (i) photosynthetic Ce6‐contained cyanobacteria were fabricated via internalization of Ce6 into cyanobacteria cells; (ii) 3D CaCO_3_‐PCL scaffolds were prepared using 3D‐printing technology; (iii) the final therapeutic platform were constructed by decorating Ce6‐contained cyanobacteria onto 3D CaCO_3_‐PCL scaffolds. As shown in Figure 10B, the engineered biohybrid scaffolds generated O_2_ upon red light irradiation (660 nm) due to photosynthesis by cyanobacteria, which subsequently activated Ce6 to produce ^1^O_2_ for highly effective PDT. Additionally, the generated O_2_ and the gradually degrading CaCO_3_‐PCL scaffolds promoted bone regeneration following tumor elimination. This study offers an insightful paradigm to utilize engineered photoresponsive biohybrids for enhanced tumor therapy and subsequent prompted tissue regeneration.

## 5 CONCLUSION AND PERSPECTIVES

Engineered photoresponsive biohybrids are a new type of biomaterials to improve the tumor therapeutic efficacy and overcome the shortcomings in current tumor phototherapy. Here, we outlined the various biomimetic properties, augmented photosensitive abilities, and multiple biotherapeutic functions of engineered photoresponsive biohybrids. We classified the engineered biohybrids as biomolecules‐associated, cell membrane‐based, eukaryotic cell‐based, bacteria‐based, and algae‐based photoresponsive biohybrids, which could be applied in various therapeutic modes, such as PTT, PDT, synergistic therapy, and tumor therapy combined with tissue regeneration. Such engineered biohybrid materials could become promising biomimetic platforms for diverse cancer therapeutic applications.

Despite many efforts on the developments of engineered photoresponsive biohybrids over the past decade, most of these photoresponsive biohybrids designed for tumor therapy are still in its infancy, and commercialization and clinical applications can be achieved. The following major challenges or obstacles should be well considered and require significant development. A key issue is the lack of simple protocols for the preparation of homogeneous functionalities. Current methods rely on complex or multi‐step operations, which are difficult to replicate in clinical settings. In addition, novel high‐performance PS should be exploitered instead of relying on building blocks from existing materials. Furthermore, although the biocompatibility and application potential of engineered photoresponsive biohybrids have been demonstrated for cells in laboratory settings, the long‐term biosafety of these biohybrids needs to be investigated for clinical settings. We believe that the above challenges can be addressed in the near future and thus facilitate the emergence of novel biohybrid designs with better improved antitumor performance and practical cancer therapeutical applications.

## AUTHOR CONTRIBUTIONS

Xiaocheng Wang: Conceptualization; Investigation; Methodology; Funding acquisition; Project administration; Resources; Writing‐original draft; Writing‐review & editing. Yazhi Sun: Investigation; Methodology; Formal analysis; Writing‐review & editing. Daniel Wangpraseurt: Conceptualization; Investigation; Methodology; Supervision; Writing‐review & editing.

## CONFLICT OF INTEREST STATEMENT

The authors declare no conflict of interest.

**Glossary**
Photodynamic therapy
: Photodynamic therapy (PDT) is a form of phototherapy involving light and a photosensitizing chemical substance, used in conjunction with molecular oxygen to elicit cell death.
Photothermal therapy
: Photothermal therapy (PTT) refers to efforts to use electromagnetic radiation (most often in infrared wavelengths) for the treatment of various medical conditions, including cancer. This approach is an extension of photodynamic therapy, in which a photosensitizer is excited with specific band light. This activation brings the sensitizer to an excited state where it then releases vibrational energy (heat), which is what kills the targeted cells.
Photothermal conversion agents
: Photothermal transduction agents (PTAs) are used in PTT to convert light energy into heat energy.
Photosensitizers
: Photosensitizers are molecules, which absorb light and transfer the energy from the incident light into another nearby molecule.
Reactive oxygen species
: In chemistry, reactive oxygen species (ROS) are highly reactive chemicals formed from diatomic oxygen (O_2_). Examples of ROS include peroxides, superoxide, hydroxyl radical, singlet oxygen, and alpha‐oxygen.
Engineered photoresponsive biohybrids
: Artificially engineered photoresponsive materials consisting of a bioactive and a structural component. The bioactive part of the biohybrid could consist of cells or bioactive molecules, and the structural part could also be of either biological or non‐biological origin.
Enhanced permeability and retention
: The enhanced permeability and retention (EPR) effect is a controversial concept by which molecules of certain sizes (typically liposomes, nanoparticles, and macromolecular drugs) tend to accumulate in tumor tissue much more than they do in normal tissues.
Endocytosis
: A cellular process in which substances are brought into the cell. The material to be internalized is surrounded by an area of cell membrane, which then buds off inside the cell to form a vesicle containing the ingested material.
Biomineralization
: A process by which living organisms produce minerals, often to harden or stiffen existing tissues.
Bioorthogonal reaction
: Refers to any chemical reaction that can occur inside of living systems without interfering with native biochemical processes.
Theragnostic
: A treatment strategy that combines therapeutics with diagnostics.
